# Intrahepatic Fat Content and Markers of Hepatic Fibrosis in Obese Children

**DOI:** 10.1155/2016/4890974

**Published:** 2016-02-04

**Authors:** Wei Wu, Hongxi Zhang, Xiaoqin Xu, Ke Huang, Junfen Fu

**Affiliations:** Children's Hospital, Zhejiang University School of Medicine, 57 Zhugan Xiang, Hangzhou, Zhejiang 310003, China

## Abstract

*Aim.* We evaluated both direct and indirect hepatic fibrosis markers in obese children and their relationship with intrahepatic fat (IHF) content. We also aimed to investigate the possible roles of IHF and fibrosis markers in metabolic syndrome (MS).* Methods.* 189 obese children were divided into simple obese (SOB), simple steatosis (SS), and nonalcoholic steatohepatitis (NASH) groups according to their IHF and blood alanine transaminase (ALT) levels. They were also scored for the MS components. IHF was assessed as a continuous variable by proton magnetic resonance spectroscopy (1H-MRS). In addition, 30 nonobese children were enrolled as controls and their direct hepatic fibrosis markers and IHF were assessed.* Results*. Age was related to IHF, NFS, and FIB-4. Both NFS and APRI were related to IHF more significantly than the direct markers. In the estimation of liver function impairment, indirect markers had greater AUROC than direct markers. In MS, IHF and all the fibrosis markers showed similar AUROC.* Conclusions*. Both direct and indirect markers played a valuable role in evaluating MS. Indirect markers were more effective in distinguishing fatty hepatitis. Age is an important factor underlying hepatic steatosis and fibrosis even in children.

## 1. Introduction

Approximately, 12% of children and adolescents in China are overweight according to the latest report [1]. The prevalence of overweight is even higher in big cities; for example, in Shanghai, 49.1% of boys and 30.8% of girls were reported to be overweight [2]. In parallel with the prevalence of obesity, nonalcoholic fatty liver disease (NAFLD), which encompasses a spectrum of conditions ranging from simple steatosis to steatohepatitis, fibrosis, and cirrhosis, represents one of the leading causes for chronic liver disease [3]. Patients with simple steatosis may have benign prognosis, whereas those with NAFLD may develop progressive liver disease [4]. However, the pathogenesis and metabolic effects of ectopic fat accumulation within the liver are still obscure. The mechanism of transition from simple steatosis [5] to fibrosis and the associated factors are also poorly characterized. Among the many tests, liver biopsy is considered to be the gold standard for NAFLD diagnosis. However, due to its invasive nature and cost, it is not appropriate for pediatric screening. Furthermore, with only very small part of the liver mass examined, it can result in sampling error and selection bias. The B-mode ultrasound for testing hepatic steatosis is noninvasive and qualitative but not helpful for quantitative analysis. Its accuracy in predicting the presence and severity of hepatic steatosis and mortality [6] is poor.

Recently, proton magnetic resonance spectroscopy (1H-MRS) has been found to be a sensitive and noninvasive method to measure hepatic triglyceride content [7, 8]. The reproducibility of the test in patients with higher hepatic triglyceride content was also found to be higher [9].

Although 30% of the obese population is at risk of NAFLD, only 3% to 5% may have NASH and 1% to 2% may have progressive liver fibrosis [10]. Therefore, early NASH staging of liver fibrosis is crucial for management and prognostication.

There are two types of hepatic fibrosis markers. Indirect markers reflecting altered hepatic function include alanine transaminase (ALT), aspartate transaminase (AST), albumin and NAFLD fibrosis score (NFS), aspartate transaminase-to-platelet ratio index (APRI), and fibrosis index based on the 4 factor (FIB-4) score. Direct markers reflecting extracellular matrix (ECM) turnover include hyaluronic acid (HA), type IV collagen (CIV), procollagen type III (PCIII), and laminin (LN). These direct markers reflect different stages of hepatic fibrosis [11].

Intrahepatic fat (IHF) assessment by 1H-MRS combined with both direct and indirect hepatic fibrosis markers in Chinese obese children has not been investigated until now. The present study was undertaken to explore (1) the magnitude of hepatic fat content in severely obese adolescents, (2) the correlation between IHF content and liver fibrosis indicators, and (3) the impact of IHF and liver fibrosis on glucose homeostasis and metabolic syndrome (MS).

## 2. Materials and Methods

### 2.1. Subjects

One hundred and eighty-nine obese children aged between 6 and 16 years with a BMI > 95th percentile and waist circumference (WC) larger than 90th percentile for their age and sex (waist-to-height hip ratio >0.48 for males and >0.46 for females according to the consensus of Chinese pediatric endocrine society, Chinese pediatric cardiovascular disease society, and Chinese child health society, [12]) were recruited to the study in the Endocrinology Department. The percentile of BMI was based on the data from Chinese children and adolescents [13]. Children with known endocrine diseases, hereditary diseases, viral hepatitis, and other chronic or infectious diseases were excluded. Pubertal stage was assessed according to the Tanner scale; 77 were prepubertal and 112 were pubertal. Obese children were further divided into three groups, simple obese (SOB) group with IHF < 5% and normal ALT, simple steatosis (SS) group with IHF > 5% and normal ALT, and the NASH group with IHF > 5% and ALT > 50, according to the lab reference standard for impaired liver function. Every child was scored for MS according to the definition of International Diabetes Federation [14]. MS in children aged between 6 and 16 years was defined as central obesity (defined as waist circumference larger than 90th percentile) and at least two of the following MS components: (1) impaired fasting blood glucose level (>5.6 mmol/L on the oral glucose tolerance test) or type 2 diabetes mellitus, (2) increased blood pressure >130 mmHg systolic or >85 mmHg diastolic, (3) high serum triglyceride (TG) level ≥150 mg/dL (1.7 mmol/L), or (4) decreased serum high-density lipoprotein cholesterol <40 mg/dL (1.03 mmol/L). The patient was assigned 1 point score in the presence of any of the MS components. The total scores were added for each patient, which ranged from 0 to 4. Since all the obese children included in our study already met the diagnostic criteria for central obesity, those who scored ≥2 (with at least 2 components of MS) were in the MS group and the rest were in the non-MS group. Thirty healthy nonobese children aged 8 to 14 years attending the Department of Child Care for health examination were recruited as the control group. Written informed consent of parents was obtained. The study protocol was approved by the Medical Ethics Committee of The Children's Hospital of Zhejiang University School of Medicine.

### 2.2. Anthropometric Measurements

Body weight was measured to the nearest 0.1 kg with only underclothing, and height was measured to the nearest 0.1 cm without socks and shoes. Waist circumference was measured at the narrowest point of the waist and hip circumference was measured at the widest point of the hip. Blood pressure was measured in a quiet sitting position four times per subject over a 2-hour period and the mean of all values was calculated. Body mass index (BMI) and ratio of waist-to-hip circumference (WHR) were also calculated.

### 2.3. Hepatic 1H-MRS

1H-MRS was performed at rest and when patients were in supine position using 1.5-T whole body scanner (Magnetom Avanto, Siemens Healthcare, Erlangen, Germany) using a conventional circular superficial coil (C1-coil). The coronal, sagittal, and transverse images of the liver were obtained for spectroscopic volume of interest (VOI) localization in all patients. A 2 cm^3^ VOI was positioned within the right lobe, avoiding major blood vessels, intrahepatic bile ducts, and the lateral margin of the liver. Voxel shimming was executed to optimize the homogeneity of the magnetic field within the specific VOI. Two ^1^H spectra (water and fat spectra) were collected from the hepatic parenchyma in the same prescanning conditions using a PRESS pulse sequence (TR = 1500 ms, TE = 30 ms, and Averages = 1,024) without suppression of the water signal. Areas of resonance from protons of water (4.7 ppm) and methylene groups in fatty acid chains of the hepatic triglycerides (1.3 ppm) were obtained with a time domain, nonlinear fitting routine using commercial software [15]. The relative liver fat content was obtained by dividing the peak area of the methylene groups in fatty acid chains of the hepatic triglycerides by the sum of methylene groups and water, multiplied by 100. To convert these values to absolute concentrations expressed as percentage of fat by weight of volume, we used equations validated by Longo et al. [16].

### 2.4. Analytical Determinations

An oral glucose tolerance test (OGTT) was performed by administering 1.75 g per kg of body weight (maximum 75 g) of glucose in all subjects. Glucose and insulin levels were determined in the blood sample drawn after an overnight fast and 2 h after the glucose load to obtain the OGTT 120 and insulin 120 values, respectively. Insulin resistance was assessed using the homeostasis model assessment of insulin resistance (HOMA-IR), which is calculated as insulin level (mIU/mL) × glucose level (mmol/L)/22.5.

Samples for measurement of fasting glucose (FG), fasting insulin (FI), ALT, AST, total cholesterol, and TG were obtained in the morning after an overnight fast. Serum glucose levels were measured with standard glucose oxidase method on a glucose analyzer (Beijing North Institute of Biological Technology, Beijing, China). Plasma levels of insulin were determined by radioimmunoassay (Beijing North Institute of Biological Technology, Beijing, China). TG, total cholesterol, high-density lipoprotein cholesterol (HDL), low-density lipoprotein cholesterol (LDL), apolipoprotein A1, and apolipoprotein B were measured by routine laboratory testing (Synchron Clinical System CX4; Beckman Instruments, Columbia, Maryland). HbA1c was measured by high performance liquid chromatography.

Serum markers of hepatic fibrosis, HA, CIV, PCIII, and LN, were measured using the ELISA kits (HA from Shanghai Naval Research Institute and others from Beijing North Institute of Biological Technology, Beijing, China). Indirect markers were calculated according to the published formula as follows [6]: NFS = −1.675 + 0.037 × age (years) + 0.094 × BMI (kg/m^2^) + 1.13 × impaired fasting glycemia or diabetes (yes = 1, no = 0) + 0.99 × AST/(ALT) ratio − 0.013 × PLT (10^9^/L) − 0.66 × ALB (g/dL). APRI = ([AST/upper limit of normal]/PLT [10^9^/L]) × 100. FIB-4 = (age [years] × AST [U/L])/(PLT [10^9^/L] × (ALT [U/L])^1/2^).

### 2.5. Statistical Analysis

Statistical analysis was performed using SPSS version 16.0 software (SPSS Inc., Chicago, Illinois). Quantitative data with normal distribution were expressed as mean ± SD, and variables with skewed distribution were assessed using Kolmogorov-Smirnov test. A normal distribution was obtained using a natural base logarithmic (LN) transformation of the data and expressed as median with interquartile range (IQR). One-way ANOVA was used for comparisons of three groups, and multiple testing was corrected using least significant difference (LSD) method, followed by least significant difference tests for multiple comparisons. Pearson's product moment correlation and bivariate correlation analysis were carried out to examine the association between variables. ROC analysis was used to examine the determinants of fatty hepatitis and MS. The significance level was set at *P* < 0.05.

## 3. Results

### 3.1. Comparison of Anthropometric Characteristics and Biochemical Parameters in Three Groups

Anthropometric characteristics of SOB, SS, and NASH groups are shown in Table 1. The WC, WHR, and BMI were significantly higher in SS and NASH groups compared with the SOB group indicating that obesity and visceral fat play a key role in the development of hepatic steatosis. However, these parameters were not different between SS and NASH groups suggesting that hepatitis cannot be differentiated from simple steatosis based on anthropometric measurements.

The biochemical parameters of the three groups are summarized in Table 2. Hepatic enzymes including ALT, AST, and *γ*-GT were significantly higher in the NASH group indicating liver injury. The hepatic enzymes in the SS group, although within the normal range, were higher compared with the SOB group. The blood lipid levels were not different between the SS and SOB groups. The TG and LDL levels were significantly higher and HDL levels were significantly lower in the NASH group. The uric acid levels were increased from SOB to SS to NASH groups.

Among the direct hepatic fibrosis markers, the PCIII in the NASH group was significantly higher than that in SS and SOB groups, while there was no difference in PCIII between the latter two groups. Therefore, PCIII levels indicated an impairment of liver function rather than simple steatosis. The differences of HA and CIV only existed between NASH and SOB groups. There was no difference in LN, HbA1c, insulin 120, and FG levels among the three groups. The OGTT 120 values were higher in NASH group than in SS and SOB groups. The HOMA-IR in NASH and SS groups was higher compared with the SOB group. The levels of CIV, PCIII, and LN were 55.32 ± 9.36, 23 (12.00, 36.45), and 109.78 ± 10.92 ng/mL, respectively, in the control group, which were significantly lower than those in the SOB group. There was no significant difference of HA between the control and the SOB groups. The IHF in the obese children was 6.9% (2.7%, 15.4%), which was significantly higher than the level of 0.6% (0.32%, 1.02%) in the control group.

Among the indirect hepatic fibrosis markers, NFS was not different between the three groups. However, APRI and FIB-4 levels in NASH group were significantly higher than those in the SS group.

Anthropometric characteristics and biochemical parameters between different genders were also compared. Boys had higher BMI (*P* = 0.001), WC (*P* = 0.006), HC (*P* = 0.017), and weight (*P* < 0.001) than the girls, while age and WHR were comparable suggesting that boys were more obese than girls but were not more centrally obese. Boys also had higher fasting insulin levels (*P* = 0.02) and HOMA-IR (*P* = 0.01) than girls. However, there was no significant difference in blood lipids (TG, LDL, HDL, and CHOL), liver function (ALT, AST, and *γ*-GT), IHF, and direct and indirect fibrosis markers as well as MS score between different genders.

### 3.2. Relationship between Hepatic Fibrosis Markers, IHF, and Biochemical Parameters

The relationship between hepatic fibrosis markers, IHF, and selected biochemical parameters is shown in Table 3. IHF was related to age, WC, blood pressure, and uric acid. When WC and BMI were controlled, the relationship between IHF and uric acid but not blood pressure was still significant with *R* = 0.33 and *P* < 0.001. IHF was also related to hepatic enzymes including ALT, AST, and *γ*-GT. The F insulin level, OGTT 120 level, and HOMA-IR were also related to IHF content. When WC and BMI were controlled, only OGTT 120 level maintained significant relationship with IHF (*R* = 0.23 and *P* = 0.002). Considering the fact that boys had significantly larger WC than girls (*P* = 0.01), when WC was controlled, we found no relationship between different genders and fibrosis markers along with IHF.

Among direct markers of hepatic fibrosis, both CIV and PCIII were related to ALT, AST, and *γ*-GT, even after controlling for BMI and WC. CIV and HA were related negatively to HDL after adjusting for BMI and WC. Among the glucose metabolism indicators, HbA1c was found to be related to LN and PCIII; insulin 120 was related to HA and FG was related to LN and HA. Among the indirect markers of hepatic fibrosis, all markers were significantly correlated with uric acid. Furthermore, NFS and FIB-4 correlated with age, and APRI and FIB-4 correlated with ALT, AST, and *γ*-GT. Among the glucose metabolism indicators, FG, OGTT 120, and HOMA-IR were correlated with only NFS. NFS was correlated with MS score most significantly (*R* = 0.358, *P* < 0.01). Both NFS and APRI were related to IHF more significantly than the direct markers.

**Figure 1:**
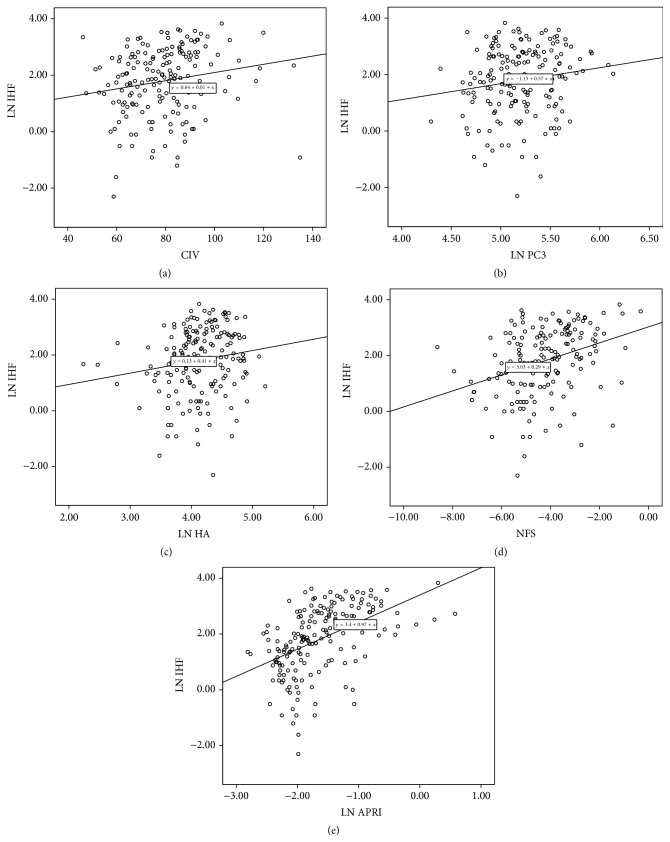
Relationship between IHF and hepatic fibrosis markers. (a) IHF and CIV: *r* = 0.179, ^*∗*^ *P* = 0.017, (b) IHF and PCIII: *r* = 0.153, ^*∗*^ *P* = 0.041, (c) IHF and HA: *r* = 0.159, ^*∗*^ *P* = 0.035, (d) IHF and NFS: *r* = 0.337, ^*∗*^ *P* < 0.001, and (e) IHF and APRI: *r* = 0.490, ^*∗*^ *P* < 0.001.  ^*∗*^means *P* < 0.05, the relationship is significant..

We also found that, in boys, testicle volume was related to FIB-4 (*r* = 0.19 and *P* = 0.03) and MS score (*r* = 0.22 and *P* = 0.01), while Tanner stage was related to the MS score (*r* = 0.23 and *P* = 0.01). However, when controlled for age, the relationships were not significant. No relationship was found between pubertal development (testosterone level, Tanner stage, and testicle volume) and hepatic changes (IHF and fibrosis markers). The relationship between IHF and hepatic fibrosis markers is shown in Figure 1. Except for LN, the other three direct markers were related significantly to IHF. Among indirect markers, NFS and APRI correlated significantly with IHF.

### 3.3. Receiver Operating Curve (ROC) Analysis of Potential Markers for NASH

**Figure 2:**
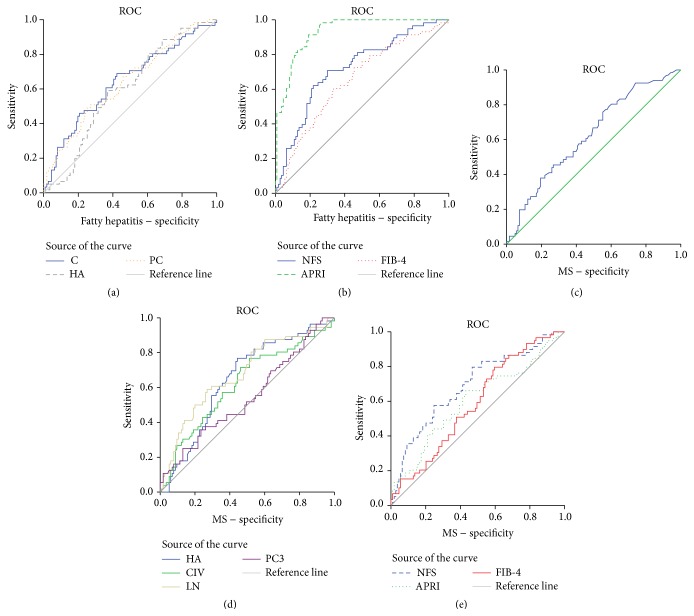
ROC in NASH and MS. In NASH, (a) CIV: area under the receiver operating curve (AUROC) = 0.641, *P* = 0.002; HA: AUROC = 0.597, *P* = 0.035; PCIII: AUROC = 0.638, *P* = 0.003; (b) NFS: AUROC = 0.722, *P* = 0.041; APRI: AUROC = 0.930, *P* = 0.0318; FIB-4: AUROC = 0.653, *P* = 0.044. In MS, (c) IHF had an AUROC of 0.639 with *P* = 0.003; (d) CIV: AUROC = 0.62, *P* = 0.01; LN: AUROC = 0.679, *P* < 0.001; HA: AUROC = 0.64, *P* = 0.002; PCIII: AUROC = 0.543, *P* = 0.36; (e) NFS: AUROC = 0.690, *P* = 0.043; APRI: AUROC = 0.601, *P* = 0.047; FIB-4: AUROC = 0.589, *P* = 0.045.

The ROC analysis of potential markers was used to predict the diagnosis of NASH. To distinguish patients with IHF > 5 (liver steatosis), all the direct and indirect hepatic fibrosis markers had AUROC of less than 0.5. As shown in Figures 2(a)-2(b), patients with IHF > 5 and ALT > 50 (i.e., liver injury) were distinguished by CIV, HA, and PCIII levels and all the indirect markers showed AUROC greater than 0.5 with *P* < 0.05. However, the indirect markers had a greater AUROC than the direct markers.

### 3.4. Comparison of Parameters in Simple Obese and MS Children

**Figure 3:**
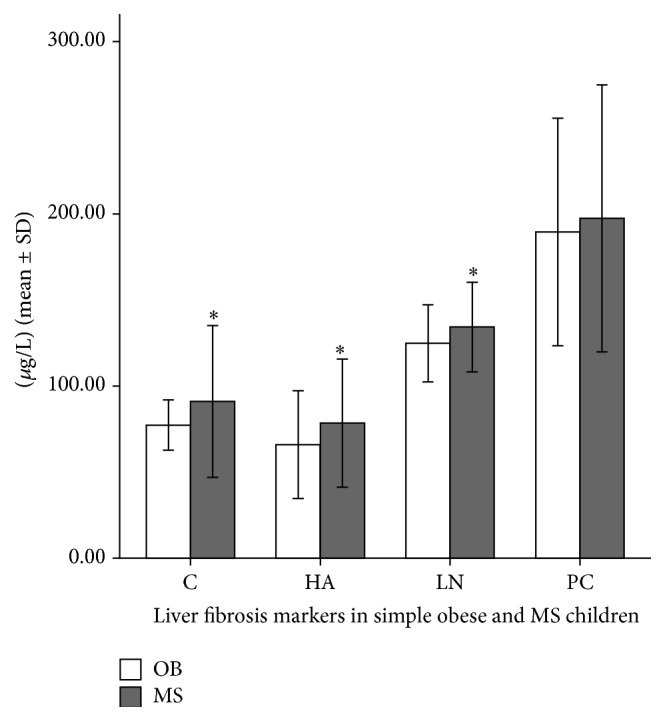
Hepatic fibrosis markers in simple obese and MS children. MS versus SOB, HA: *P* = 0.019; CIV:  *P* = 0.021; LN:  *P* = 0.013; PCIII:  *P* = 0.48.  ^*∗*^refers to *P* < 0.05 and the difference between two groups was significant. Error bars: ± 1 SD.

The hepatic fibrosis markers in simple obese and MS children are compared in Figure 3. The MS score was related significantly to HA (*r* = 0.217  and  *P* = 0.004), CIV (*r* = 0.159  and  *P* = 0.036), and LN (*r* = 0.184  and  *P* = 0.014).

**Figure 4:**
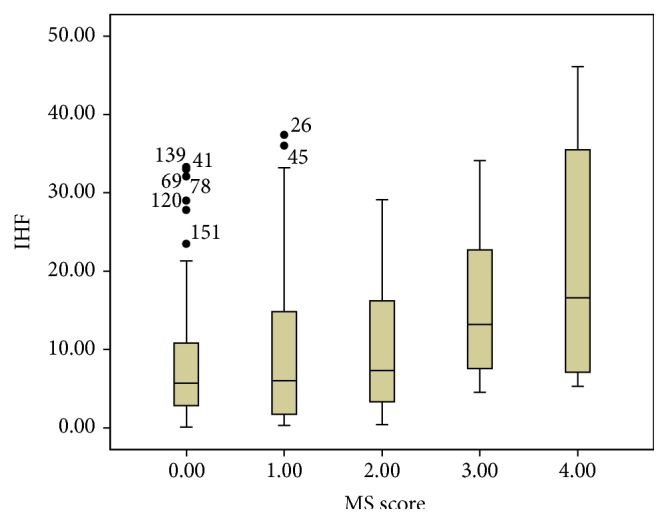
Boxplots of IHF with respect to MS score. The lower boundary of the box and whisker plot corresponds to the 25th percentile, the line within the box corresponds to the median, and the upper boundary of the box corresponds to the 75th percentile. The whiskers extend to the most extreme data point, which is no more than 1.5 times the interquartile range from the box.

The relationship between IHF and MS score was found to be significant by Spearman's test (*r* = 0.209 and *P* = 0.007). Values of IHF in relation to MS score are shown in Figure 4.

The values of MS, IHF, CIV, LN, and HA had AUROC greater than 0.5 with *P* < 0.01 indicating their role in distinguishing MS in obese children, as shown in Figures 2(c)–2(e).

Among the four direct hepatic fibrosis markers, HA, CIV, and LN enabled the estimation of MS. CIV, HA, and PCIII were also related to IHF and useful in identifying fatty hepatitis. IHF was also related to MS score, suggesting that liver steatosis played a role in MS development.

## 4. Discussion

To the best of our knowledge, this study is the first to evaluate the quantitative correlation between direct and indirect hepatic fibrosis markers and hepatic fat content measured by 1H-MRS in obese children.

A major strength of the study is the inclusion of a large group of consecutively recruited children with robust IHF assessment. Another major strength relates to the introduction of both direct and indirect hepatic fibrosis markers in obese children, with the direct markers reflecting the ECM metabolism of the liver. The limitations include failure to obtain liver biopsy and use of serum ALT in defining NASH, which is a suboptimal surrogate.

The direct hepatic fibrosis markers reflecting the ECM metabolism correlate with dynamic changes in fibrogenesis and resolution of fibrosis. Increased synthesis and decreased degradation of HA are accompanied by accelerated ECM synthesis and attenuation of ECM degradation. HA is considered as a valuable tool to assess necrosis of liver injuries in parallel with liver biopsy [17] and a good indicator of hepatic fibrosis [18] in children [19]. CIV and LN are mainly present in the basement membrane and are useful in the evaluation of pathological changes during liver fibrosis [20]. PCIII reflects ongoing collagen formation in the liver [21]. The role of PCIII and LN in estimating hepatic fibrosis has been reported [22].

On the other hand, indirect markers are calculated mostly based on the age, BMI, and liver function indicators including albumin and ALT, AST, and indicators of inflammation such as platelets. Recent studies indicated that only the top 5% of the biomarkers such as AST, ALT, APRI, and FIB-4 were consistent with the absence of advanced liver disease but poorly related to the presence of advanced disease [23]. FIB-4 index (>1.659) and NAFLD fibrosis score (>0.735) were found to have higher sensitivity and specificity for the prediction of advanced fibrosis [24].

In our study, direct markers including CIV, HA, and PCIII correlated with IHF and distinguished fatty hepatitis. A previous study also indicated CIV and PCIII as the only markers of histological diagnosis of NASH [25]. Another study reported that the detection of severe fibrosis (>F3) was excellent and the detection of fibrosis was modest by these markers [26]. The smaller AUC for discriminating fatty hepatitis based on these markers in our study compared with previous studies may be attributed to younger age and obesity during the early stages of fibrosis compared with the adult population. Age correlated with IHF and ALT significantly in our study. The best cutoff values of 148.8 ng/mL for HA and 292.5 ng/mL for LN [27] were higher than the data in our study.

The indirect markers were found to be better in discriminating fatty hepatitis from hepatic steatosis than direct markers, a fact which was especially true in case of APRI (AUROC 0.93). In the evaluation of MS, all the indicators were consistent with an AUROC of around 0.6.

The IHF is also related to MS score. IHF levels are comparable among patients with MS score from 0 to 2. The patients who scored greater than score 2 have a higher IHF than those who scored less than 2. Even in MS patients, those scoring 4 had higher IHF compared to those scoring 3. Hence, fatty liver was recognized as a novel component of MS [28].

Diabetes mellitus is a significant risk factor for advanced fibrosis in patients with NAFLD [29]. Insulin resistance is also a major factor associated with NAFLD and significant liver fibrosis [30, 31]. NAFLD itself was a multisystem disease and associated with factors that mediate interindividual variations in the development of extrahepatic manifestations including type 2 diabetes mellitus [32]. Our study explored the relationship between glucose metabolism-related factors and hepatic fibrosis markers simultaneously. OGTT 120 was related to IHF independent of BMI and WC. HbA1c was related to LN and PCIII; insulin 120 was related to HA and fasting glucose was related to LN and HA. NFS was related to fasting glucose, OGTT 120, and HOMA-IR. These facts demonstrate that the hepatic fibrosis markers were related to glucose and insulin metabolism indicators as well. Therefore, liver steatosis and fibrosis were possibly involved in glucose metabolism and insulin resistance even in children.

WC was related to IHF and APRI. The relationship between IHF and WC was not gender-based. However, APRI and WC were related in girls. Anthropometric measurements were associated with the severity of NAFLD based on gender, especially in women with larger WC showing a greater likelihood of liver injury [33]. Age is also an important factor related to liver injury. Our data showed that age is related to IHF, NFS, and FIB-4 similar to an earlier study, which indicated that age was related to liver fibrosis [33]. The uric acid values increased from SOB group to SS group and further to NASH group. Hyperuricemia has been found commonly in NAFLD [34], mediated by xanthine oxidase [35]. Uric acid inducing fat accumulation via generation of endoplasmic reticulum stress in hepatocytes has been reported [36].

The study findings reflect potential bias, with the boys constituting two-thirds of the total sample. However, gender distribution of obese children in the present study was similar to an early report from China. In a sample of 4,094 participants, 568 boys (26.7%) and 326 girls (16.6%) were found to be overweight or obese [37]. Other studies in China also reported that boys were more often obese than girls [38–40]. The male-specific prevalence of obesity might be related to gender differences in feeding and perceptions of body image. In China, boys are placed at a higher value and considered strong, and big body is considered healthy, while girls are more concerned about their body shape, which also explains why boys were more obese than girls in our study. No significant relationship was found between pubertal developments (testosterone level, Tanner stage, and testicle volume) and hepatic changes (IHF and fibrosis markers). The results were different from previous studies in adults and rabbit models [13, 41], which indicated that testosterone plays a protective role in NAFLD. This difference could be attributed to the fact that 45% of the boys were prepubertal (testicle volume < 4 mL) and 72% of the boys had a testosterone level < 20 mmol/L, which is prepuberty level.

In conclusion, indirect hepatic fibrosis markers were better than direct markers in discriminating fatty hepatitis. Both direct and indirect markers were useful in evaluating MS. Age is an important factor for hepatic steatosis and fibrosis even in children. Boys were more obese (but not more centrally obese) than girls. Waist circumference was related to liver steatosis and fibrosis in both genders.

## Figures and Tables

**Table 1:** Comparison of anthropometric measurements.

Groups	SOB	SS	NASH
*N* (F/M)	75 (37/38)	49 (11/38)	65 (16/49)

Age (y)	10.45 ± 2.45 (6–16)	10.56 ± 2.15 (6–15)	11.12 ± 2.05^*∗*^ (6–16)

Height (cm)	144.98 ± 13.20 (119–168)	147.42 ± 11.82^*∗*^ (116–166)	150.42 ± 12.07^*∗*^ (118–177)

Weight (kg)	57.63 ± 17.30 (31–110)	63.43 ± 14.97^*∗*^ (33.5–107)	65.76 ± 16.5^*∗∗*^ (34.5–128)

WC (cm)	84.19 ± 10.40 (66–115)	89.88 ± 8.97^*∗∗*^ (72–112)	91.52 ± 10.49^*∗∗*^ (68–125)

HC (cm)	91.11 ± 10.62 (73–120)	93.79 ± 8.74 (75.5–122)	93.63 ± 15.45 (71.5–131)

WHR	0.92 ± 0.06 (0.77–1.08)	0.96 ± 0.06^*∗∗*^ (0.80–1.07)	0.96 ± 0.05^*∗∗*^ (0.84–1.07)

BMI (kg/m^2^)	26.83 ± 4.01 (20.35–40.30)	28.80 ± 3.75^*∗∗*^ (23–40.67)	28.64 ± 3.99^*∗∗*^ (20–43.8)

SBP (kpa)	114.35 ± 14.21 (90–151)	119.51 ± 13.00^*∗*^ (89–148)	118.87 ± 14.17 (90–153)

DBP (kpa)	67.25 ± 7.80 (48–91)	71.20 ± 7.61^*∗*^ (56–86)	69.24 ± 7.80 (55–95)

WC: waist circumference; WHR: ratio of waist circumference and hip circumference; BMI: body mass index; SBP: systolic blood pressure; DBP: diastolic blood pressure.

VS SOB, ^*∗*^ *P* < 0.05; ^*∗∗*^ *P* < 0.01.

Quantitative data are expressed as the mean ± SD and the ranges are indicated in parentheses.

**Table 2:** Comparison of biochemical parameters.

Parameters	SOB (*n* = 75)	SS (*n* = 49)	NASH (*n* = 65)
ALT (U/L)	18 (14–23) (4–114)	37^*∗∗*^ (29.5–43.5) (17–50)	96^*∗∗*##^ (69.5–137) (51–370)

AST (U/L)	23 (20–26) (12–74)	28^*∗*^ (24.5–32) (18–102)	51^*∗∗*##^ (41–75.5) (27–293)

*γ*-GT (U/L)	16 (13–21) (7–71)	23^*∗∗*^ (19.5–28) (2–71)	42^*∗∗*##^ (28.5–65) (17–138)

TG (mmol/L)	0.95 (0.82–1.39) (0.39–3.69)	1.33 (0.94–1.77)^*∗*^ (0.4–3.86)	1.43 (0.93–1.80)^*∗∗*^ (0.65–4.69)

CHOL (mmol/L)	4.28 ± 0.71 (3.09–6.25)	4.32 ± 0.86 (2.54–6.55)	4.59 ± 0.98^*∗*^ (1.14–7.73)

HDL (mmol/L)	1.27 ± 0.37 (0.77–2.71)	1.17 ± 0.31 (0.58–1.79)	1.05 ± 2.63^*∗∗*^ (0.57–1.66)

LDL (mmol/L)	2.32 ± 0.53 (1.07–3.91)	2.44 ± 0.64 (1.19–4.09)	2.61 ± 0.55^*∗∗*^ (1.3–3.85)

Uric acid (*µ*mol/L)	346.02 ± 80.3 (201.9–586.4)	392.14 ± 74.05^*∗∗*^ (253.1–568.5)	429.38 ± 90.72^*∗∗*#^ (226.7–673.6)

HA (ng/mL)	59.56 (40.22–81.6) (16–183)	60.14 (49.7–88.53) (9–130)	71.7^*∗*^ (53.04–94.02) (16–166)

CIV (ng/mL)	78.79 ± 27.77 (48–135)	82.20 ± 32.40 (53–118)	85.49 ± 27.76^*∗*^ (46–132)

PCIII (ng/mL)	173.14 (133.94–217.14) (73–299)	161.5 (138.44–198.20) (81–368)	195.1^*∗∗*##^ (153.75–258.58) (106–462)

LN (ng/mL)	128.90 ± 21.17 (67–195)	128.99 ± 17.55 (73–178)	126.34 ± 30.71 (14–214)

NFS	−5.57 ± 1.34 (−7.94–−1.11)	−5.50 ± 1.33 (−8.63–−0.94)	−5.29 ± 1.23 (−6.45–−0.32)

APRI	0.125 (0.10–0.16) (0.06–0.41)	0.16 (0.14–0.18)^*∗*^ (0.08–0.7)	0.33 (0.23–0.45)^*∗∗*##^ (0.15–1.78)

FIB-4	0.17 (0.12–0.23) (0.08–0.56)	0.15 (0.13–0.17) (0.07–0.71)	0.19 (0.16–0.26)^##^ (0.07–0.64)

HbA1c (%)	5.76 ± 0.41 (4.9–7.0)	5.90 ± 0.49 (4.8–6.9)	5.85 ± 0.39 (4.9–6.8)

Insulin 0 (*µ*IU/mL)	16.10 (12.38–22.38) (0.8–46.1)	19.9 (13.85–31.15)^*∗*^ (1.3–64.3)	21.60 (14.40–30.30)^*∗*^ (0.9–56.9)

Insulin 120 (*µ*IU/mL)	53.10 (30.10–81.40) (1.9–174.2)	73.10 (29.15–119.85) (2.4–300)	78.80 (29.50–144.65) (2.1–300)

OGTT 120 (mmol/L)	6.50 (5.10–11.10) (5.1–11.1)	6.70 (6025–7.40) (5.4–10)	7.30 (6.40–7.90)^*∗∗*#^ (5–18.8)

FG (mmol/L)	5.40 (5.10–5.70) (4.3–7.7)	5.40 (4.40–8.90) (4.4–8.9)	5.50 (5.25–5.75) (4.1–8)

HOMA-IR	3.79 (2.85–5.08) (0.18–10.65)	4.77 (3.37–7.06)^*∗*^ (0.31–24.29)	5.03 (3.58–7.48)^*∗*^ (0.23–15.17)

Compared with SOB, ^*∗*^ *P* < 0.05 and ^*∗∗*^ *P* < 0.01; compared to SS, ^#^ *P* < 0.05 and ^##^ *P* < 0.01.

Quantitative data with normal distribution are expressed as the mean ± SD, and variables with skewed distribution are presented as median with interquartile range. The ranges of all the parameters were listed in the bracket.

**Table 3:** Relationship between hepatic fibrosis markers, IHF, and biochemical parameters.

*r*	CIV	LN	LN HA	LN PCIII	LN IHF	NFS	LN APRI	LN FIB-4
Age	−0.011	−0.012	0.011	−0.022	0.164^*∗*^	0.288^**∗∗**^	0.126	0.525^**∗∗**^
WC	0.055	−0.089	0.059	0.056	0.326^**∗∗**^	−0.022	0.166^*∗*^	−0.111
SBP	0.024	−0.09	−0.02	0.055	0.147^*∗*^	0.189^*∗*^	0.122	0.207^**∗∗**^
Uric acid	0.135	−0.066	0.106	0.163^*∗*^	0.414^**∗∗**^	0.213^**∗∗**^	0.259^**∗∗**^	0.173^*∗*^
HDL	−0.214^**∗∗**^	−0.067	−0.201^**∗∗**^	−0.049	−0.296^**∗∗**^	−0.134	−0.276^**∗∗**^	−0.109
LDL	−0.013	−0.046	0.046	0.042	0.170^*∗*^	0.124	0.136	0.042
LN TG	−0.006	0.047	0.091	0.122	0.274^**∗∗**^	0.092	0.112	−0.075
LN ALT	0.224^**∗∗**^	0.015	0.145	0.233^**∗∗**^	0.725^**∗∗**^	0.040	0.780^**∗∗**^	0.156^**∗∗**^
LN AST	0.315^**∗∗**^	0.107	0.138	0.312^**∗∗**^	0.545^**∗∗**^	0.059	0.888^**∗∗**^	0.398^**∗∗**^
LN *γ*-GT	0.195^**∗∗**^	−0.001	0.163^*∗*^	0.246^**∗∗**^	0.586^**∗∗**^	0.059^*∗*^	0.632^**∗∗**^	0.201^**∗∗**^
LN F insulin	−0.068	−0.078	0.098	0.042	0.164^*∗*^	0.145	−0.104	−0.083
LN insulin 120	−0.04	−0.089	−0.198^**∗∗**^	−0.08	0.091	0.033	−0.022	0.017
Fasting glucose	0.065	0.190^*∗*^	0.176^*∗*^	0.031	−0.013	0.376^**∗∗**^	0.104	0.097
LN OGTT 120	0.042	0.09	0.131	0.035	0.247^**∗∗**^	0.184^*∗*^	0.207	0.107
HbA1c	−0.041	0.207^**∗∗**^	−0.055	0.190^*∗*^	0.117	−0.001	0.094	0.121
LN HOMA-IR	−0.057	−0.05	0.123	0.045	0.163^*∗*^	0.198^*∗*^	−0.083	−0.065
MS score	0.146	0.171^*∗*^	0.172^*∗*^	0.063	0.230^**∗∗**^	0.358^**∗∗**^	0.234	0.165

Pearson's correlation tests were performed. The variables without normal distribution were natural base logarithmic (LN) transformed to normal distribution before analysis. ^*∗*^ *P* < 0.05; ^*∗∗*^ *P* < 0.01.
